# Revealing the Cognitive Neuroscience of Belief

**DOI:** 10.3389/fnbeh.2022.926742

**Published:** 2022-07-18

**Authors:** Michael H. Connors, Peter W. Halligan

**Affiliations:** ^1^Centre for Healthy Brain Ageing, University of New South Wales, Sydney, NSW, Australia; ^2^School of Psychology, Cardiff University, Cardiff, United Kingdom

**Keywords:** belief, belief formation, cognition, cognitive neuropsychiatry, credition, delusion, psychosis

## Introduction

Beliefs are convictions about what we accept as true. They provide the fundamental framework that we use to understand and engage meaningfully with the world. They also serve important social functions, such as in identity, relationships, and group coordination. Despite their personal and social significance, beliefs as psychological constructs have been largely neglected in empirical studies until recently. In previous work, we noted how studying delusion—defined as a pathological form of belief—provide a unique window to better understanding belief. Drawing on this approach and other psychological disciplines, we proposed a number of core functions and properties of belief. We also outlined a provisional five-stage cognitive model of belief formation. This paper provides an overview and discusses the implications of this account for psychology and cognitive neuroscience. In particular, we suggest that the five-stage model offers a tentative conceptual structure that could help foster future interdisciplinary research and render beliefs more tractable for scientific study.

## Studying Belief

The notion of belief is frequently invoked and indeed assumed in everyday life and across all academic and clinical disciplines. Clarifying the construct is critical, in particular, for cognitive psychology given its responsibility to characterize the mental processes underpinning how we, as self-embodied individuals, understand and engage with others and our physical environment (Connors and Halligan, 2015). Despite this, empirical research and theoretical discussions within psychology remain limited. This has likely been driven by difficulties operationalizing this ubiquitous and nebulous term. Philosophical debates about the nature of belief continue (Schwitzgebel, 2010); folk conceptions vary (Pechey and Halligan, 2012b); and cognitive accounts have not been available until recently. Such issues, however, can be overcome. Philosophical debates and folk conceptions need not preclude empirical study (Bell et al., 2006) and recent theoretical developments offer greater clarity around research directions (Connors and Halligan, 2020).

A related challenge for research has been the inherent complexity of belief. Beliefs exist within broader networks of related beliefs (Quine and Ullian, 1970), making discrete beliefs difficult to study in isolation. Beliefs also interact with many lower-level cognitive processes, such as attention, perception, and memory. Given such close inter-relationships with automatic cognitive processes, Fodor (1983) argued that belief could not be decomposed into discrete independent subcomponents (modules) or localized neuroanatomically, limiting the viability of scientific study. These pessimistic accounts have been challenged over recent decades. While some suggest that it is premature to accept that belief is non-modular (Coltheart, 2017), others note that, even if this turns out to be the case, it need not follow that scientific study is impossible (Murphy, 2019). Scientific methods have been effectively applied to many complex systems and other forms of higher-order cognition, suggesting that analogous methods could be developed for the unique subject matter (Connors and Halligan, 2020).

One method that offers promise when addressing these practical challenges is the study of delusions. Delusions offer salient examples of pathological belief and often reflect relatively circumscribed dysfunction within an individual's cognitive system. By careful study, one can identify specific contributory factors and their impact. Such study, in turn, can offer insights into the cognitive processes involved in other delusions and belief formation more generally (Connors and Halligan, 2015, 2017, 2020). This approach is known as cognitive neuropsychiatry—a discipline that seeks to explain neuropsychiatric symptoms in terms of disruptions or damage to normal cognitive processes (Halligan and David, 2001).

The approach can be briefly illustrated when applied to the Capgras delusion—a false belief that a familiar person has been replaced by an impostor. Research has found that multiple patients with this delusions have a deficit in their autonomic response to familiar faces (Ellis et al., 1997). This deficit could plausibly lead to an unexpected sense of unfamiliarity around others and hence a conclusion that a familiar person is an impostor (Ellis and Young, 1990). Similar accounts can be offered for the content of other delusions, reflecting the more general point that delusions may arise from an attempt to explain unusual sensory data. Some patients, however, experience anomalous data without developing a delusion. This suggests the need for another factor—such as a deficit in belief evaluation (Coltheart et al., 2011)—to explain why some patients accept the delusion and others do not. Whilst aspects of this account remain subject to discussion, the example highlights the broader potential to examine cognitive processes contributing to delusional belief.

## A Five-Stage Account

Based on evidence from delusions and other psychological disciplines, we previously identified several core functions of beliefs. These include providing a consistent representation of our social and physical world; offering an explanatory framework; coordinating lower-level cognitive processes; and facilitating social functions, such as identity, relationships, and group coordination (Connors and Halligan, 2015). We also identified a range of dimensions of belief, such as their origins, conviction, stability, conscious awareness, and impact.

**Figure 1:**
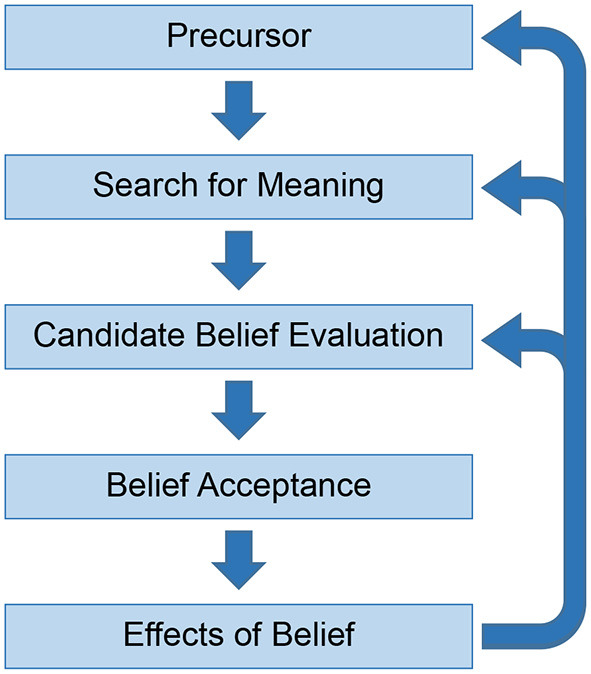
Five stages of belief formation.

Against this background, we outlined a tentative five-stage cognitive model of belief formation (Connors and Halligan, 2015, 2017, 2020). This noted that beliefs are likely to arise in response to a precursor, a distal trigger for the belief's content (Figure 1). Between the precursor and the belief, at least two intermediate stages are needed: firstly, ascribing meaning to the precursor and, secondly, evaluating the proposed meaning in terms of whether it meets criteria for belief. After a belief is formed, a fifth stage is the effect the belief then has on subjective experience and other cognitive processes, including other beliefs. This overall account is not committed to modularity and individual stages are likely underpinned by a wide range of automatic and unconscious cognitive processes (Oakley and Halligan, 2017). It is nevertheless possible to characterize these broad stages in more detail.

The first stage is a precursor that operates as a distal trigger for a belief's content. This could involve sensory input, particularly if unexpected or otherwise salient. It could, however, also take other forms, such as communication from trusted others. Indeed, many beliefs, including pathological forms, appear to arise from accepting social communicated ideas, rather than direct sensory experience (Sperber, 2009). A further form of precursor may be introspection on memories, imagery, or pre-existing beliefs, which can likewise occur without immediate sensory input. For delusions, source monitoring errors—failures to identify the origins of internally-generated thoughts, memories, and actions—may provide an important source of content (Johnson, 1988; Griffin and Fletcher, 2017).

The second stage is a search for meaning to interpret and explain the precursor. This draws heavily upon pre-existing beliefs and other relevant contextual information. As such, the resulting proto-beliefs can be highly personal and idiosyncratic. Interpretation is likely, in particular, to seek to preserve pre-existing beliefs for internal consistency and avoid dissonance. Interpretation is also likely to reflect particular attributional styles (habitual tendencies to explain events in certain ways); heuristics to reduce cognitive effort; emotion and mood; and social motivations (e.g., preserving a positive sense of self and maintaining relationships and group ties). Such processes shape the content of beliefs beyond what is specified by the precursor itself.

The third stage involves evaluating the proto-beliefs. This is likely based on at least two key criteria, namely observational adequacy (the degree to which the belief explains the precursor) and doxastic conservatism (consistency with pre-existing beliefs; Stone and Young, 1997; Mckay, 2012). The latter tendency is particularly important for maintaining internal consistency, so conflicting accounts are likely subjected to intense scrutiny. For delusions, disruptions in belief evaluation may give rise to implausible content by allowing hypotheses to be accepted without adequate examination. Such disruptions, however, are not necessary for all delusions. Supportive pre-existing beliefs and/or cognitive biases within the subject's own community could also allow many unusual beliefs to be accepted. Indeed, once delusions are formed, belief evaluation may serve to reject alternative, non-delusional accounts to maintain internal consistency.

The fourth stage is activation of the new belief. This will usually need to be co-located within a network of inter-related beliefs to be maintained. As already noted, beliefs vary in specific properties and multiple factors are likely to influence each of these. Of particular significance are a belief's conviction and influence on action. These features are likely to depend on similar criteria as those in belief evaluation, namely the belief's adequacy at predicting ongoing experience and congruence with other pre-existing beliefs. Both criteria, as well as a belief's salience, can vary to some degree across time and context, so it is possible that a belief's conviction and influence may similarly vary (Connors and Coltheart, 2011). Most beliefs, however, are likely to fit within a network of consistent and mutually supportive beliefs (Pechey and Halligan, 2012a; Seitz et al., 2018), so are likely to remain relatively stable.

The final stage is the impact the belief has on lower-level cognitive processes and broader subjective experience. In everyday life, beliefs are experienced as lived and typically not subject to decomposition, questioning, or reflection at the time. As representations of one's phenomenal world, beliefs strongly influence attributions and the deployment of lower-order processes, such as attention, perception, and memory, in a top-down manner. Whilst constrained by sensory data, beliefs bias cognitive processing, particularly when data are ambiguous, to align with the beliefs' predictions. Specific mechanisms remain contested, including the extent to which beliefs affect basic perception (Vetter and Newen, 2014). Nevertheless, the overall impact of beliefs on attributions and subjective experience is evident across many experimental paradigms (Hastorf and Cantril, 1954; Jones and Russell, 1980; Gilovich, 1991; Gregory, 1997; Irwin, 2009; Connors et al., 2015). As such, beliefs, including delusional forms, provide an incredibly powerful lens that shapes our experience, affecting what we attend to, perceive, remember, and consider plausible as an explanation. This, in turn provides further support for the belief and lead to the elaboration of related beliefs and broader world views.

## Implications

Our account of belief formation is admittedly preliminary and underspecified. We consider it, however, to be parsimonious and helpful when trying to explain the heterogeneity of belief, including delusions and other anomalous forms. We also believe that it has sufficient detail to guide future research. Our five-stage account highlights, in particular, how belief formation can be functionally decomposed, independent of assumptions around cognitive architecture and modularity. This has relevance to other areas of psychology, cognitive neuroscience, and many other academic disciplines. It also provides a more comprehensive account of delusions than existing cognitive accounts, which have a number of significant empirical and theoretical limitations (Connors and Halligan, 2020, 2021a,b).

Research methodologies from many disciplines are relevant when elucidating the cognitive processes implicated. While studying delusions is likely to remain important, observational research of beliefs in the non-clinical population will be needed to better define characteristics of normality and dysfunction. Strongly-held beliefs with anomalous content—such as conspiracy theories and certain religious and political beliefs—may be particularly relevant in this respect and provide insight into developmental antecedents, personality factors, neuropsychological correlates, and social dynamics (Pechey and Halligan, 2011; Douglas et al., 2017). Experimental methods that directly alter belief, including associative learning, hypnosis (Oakley and Halligan, 2013; Connors, 2015), and social influence (Cialdini, 2021), are also likely to be important in clarifying psychological mechanisms.

A final challenge involves marrying the cognitive processes of belief to the underlying neurobiology (Bell and Halligan, 2013). Recent accounts have highlighted potential neurophysiological processes involved in believing (“credition”; Seitz et al., 2018). Importantly, however, neuroimaging and other investigative techniques depend in large part on the cognitive models and behavioral tasks used (Poldrack and Farah, 2015). As such, the five-stage account provides an initial cognitive framework to guide investigation. Our account also highlights challenges establishing specificity of associations given beliefs' heterogeneous properties; frequent coalescence around shared themes; and close connections with lower-level automatic cognitive systems. Contrary to Fodor, these challenges are not necessarily insurmountable, though care will be need to be taken in experimental designs and likely require converging methodologies. Computational modeling and predictive data-driven approaches may assist, though both similarly remain limited to some extent by the overarching cognitive framework used (Poldrack and Yarkoni, 2016). Progress in cognitive neuroscience is therefore likely to remain closely linked to elucidating belief's cognitive basis. Further clarification of both promises to offer important insights into consciousness, social processes, and ourselves.

## Author Contributions

MC and PH were involved in conceptualizing, writing, and revising this paper for critically meaningful content. Both authors approved the final version.

## Funding

Publication fees were paid by Professor Rüdiger Seitz via the Volkswagen Foundation, Siemens Healthineers, and the Betz Foundation.

## Conflict of Interest

The authors declare that the research was conducted in the absence of any commercial or financial relationships that could be construed as a potential conflict of interest.

## Publisher's Note

All claims expressed in this article are solely those of the authors and do not necessarily represent those of their affiliated organizations, or those of the publisher, the editors and the reviewers. Any product that may be evaluated in this article, or claim that may be made by its manufacturer, is not guaranteed or endorsed by the publisher.

## References

[B1] Bell V. Halligan P. W. (2013). “The neural basis of abnormal personal belief,” in The Neural Basis of Human Belief Systems, eds F. Krueger and J. Grafman (Hove, UK: Psychology Press), 191–224.

[B2] Bell V. Halligan P. W. Ellis H. D. (2006). “A cognitive neuroscience of belief,” in The Power of Belief: Psychosocial Influence on Illness, Disability and Medicine, eds P. W. Halligan and M. Aylward (Oxford, UK: Oxford University Press), 3–20.

[B3] Cialdini R. B. (2021). Influence: The Psychology of Persuasion. New York, NY: HarperCollins.

[B4] Coltheart M. (2017). The assumptions of cognitive neuropsychology: reflections on Caramazza (1984, 1986). Cogn. Neuropsychol. 34, 397–402. 10.1080/02643294.2017.1324950 28514877

[B5] Coltheart M. Langdon R. Mckay R. (2011). Delusional belief. Annu. Rev. Psychol. 62, 271–298. 10.1146/annurev.psych.121208.131622 20731601

[B6] Connors M. H. (2015). Hypnosis and belief: a review of hypnotic delusions. Conscious. Cogn. 36, 27–43. 10.1016/j.concog.2015.05.015 26057405

[B7] Connors M. H. Barnier A. J. Langdon R. Coltheart M. (2015). Hypnotic models of mirrored-self misidentification delusion: a review and an evaluation. Psychol. Consciousness 2, 430–451. 10.1037/cns0000059

[B8] Connors M. H. Coltheart M. (2011). On the behaviour of senile dementia patients vis-à-vis the mirror: Ajuriaguerra, Strejilevitch and Tissot (1963). Neuropsychologia 49, 1679–1692. 10.1016/j.neuropsychologia.2011.02.041 21356221

[B9] Connors M. H. Halligan P. W. (2015). A cognitive account of belief: a tentative roadmap. Front. Psychol. 5, 1588. 10.3389/fpsyg.2014.01588 25741291 PMC4327528

[B10] Connors M. H. Halligan P. W. (2017). “Belief and belief formation: Insights from delusions,” in Processes of Believing: The Acquisition, Maintenance, and Change in Creditions, eds H.-F. Angel, L. Oviedo, R. F. Paloutzian, A. L. C. Runehov, and R. J. Seitz (Cham: Springer International Publishing), 153–165.

[B11] Connors M. H. Halligan P. W. (2020). Delusions and theories of belief. Conscious. Cogn. 81, 102935. 10.1016/j.concog.2020.102935 32334355

[B12] Connors M. H. Halligan P. W. (2021a). Delusions and disorders of self-experience. Lancet Psychiatry 8, 740–741. 10.1016/S2215-0366(21)00244-3 34358476

[B13] Connors M. H. Halligan P. W. (2021b). Phenomenology, delusions, and belief. Lancet Psychiatry 8, 272–273. 10.1016/S2215-0366(21)00027-4 33743872

[B14] Douglas K. M. Sutton R. M. Cichocka A. (2017). The psychology of conspiracy theories. Curr. Dir. Psychol. Sci. 26, 538–542. 10.1177/0963721417718261 29276345 PMC5724570

[B15] Ellis H. D. Young A. W. (1990). Accounting for delusional misidentifications. Br. J. Psychiatry 157, 239–248. 10.1192/bjp.157.2.239 2224375

[B16] Ellis H. D. Young A. W. Quayle A. H. De Pauw K. W. (1997). Reduced autonomic responses to faces in Capgras delusion. Proc. R. Soc. B Biol. Sci. 264, 1085–1092. 10.1098/rspb.1997.0150 9263474 PMC1688551

[B17] Fodor J. A. (1983). The Modularity of Mind: An Essay on Faculty Psychology. Cambridge, MA: The MIT Press.

[B18] Gilovich T. (1991). How we Know What isn't so: The Fallibility of Human Reason in Everyday Life. New York, NY: The Free Press.

[B19] Gregory R. L. (1997). Eye and Brain: The Psychology of Seeing. Princeton, NJ: Princeton University Press.

[B20] Griffin J. D. Fletcher P. C. (2017). Predictive processing, source monitoring, and psychosis. Annu. Rev. Clin. Psychol. 13, 265–289. 10.1146/annurev-clinpsy-032816-045145 28375719 PMC5424073

[B21] Halligan P. W. David A. S. (2001). Cognitive neuropsychiatry: towards a scientific psychopathology. Nat. Rev. Neurosci. 2, 209–215. 10.1038/35058586 11256082

[B22] Hastorf A. H. Cantril H. (1954). They saw a game: a case study. J. Abnorm. Soc. Psychol. 49, 129–134. 10.1037/h0057880 13128974

[B23] Irwin H. J. (2009). The Psychology of Paranormal Belief: A Researcher's Handbook. Hertfordshire: University of Hertfordshire Press.

[B24] Johnson M. K. (1988). “Discriminating the origin of information,” in Delusional Beliefs, eds T. F. Oltmanns and B. A. Maher (New York, NY: John Wiley and Sons), 34–65.

[B25] Jones W. H. Russell D. (1980). The selective processing of belief disconfirming information. Eur. J. Soc. Psychol. 10, 309–312. 10.1002/ejsp.2420100309

[B26] Mckay R. (2012). Delusional inference. Mind Lang. 27, 330–355. 10.1111/j.1468-0017.2012.01447.x

[B27] Murphy G. L. (2019). On Fodor's first law of the nonexistence of cognitive science. Cogn. Sci. 43, e12735. 10.1111/cogs.12735 31087591

[B28] Oakley D. A. Halligan P. W. (2013). Hypnotic suggestion: opportunities for cognitive neuroscience. Nat. Rev. Neurosci. 14, 565–576. 10.1038/nrn3538 23860312

[B29] Oakley D. A. Halligan P. W. (2017). Chasing the rainbow: the non-conscious nature of being. Front. Psychol. 8, 1924. 10.3389/fpsyg.2017.01924 29184516 PMC5694471

[B30] Pechey R. Halligan P. (2011). The prevalence of delusion-like beliefs relative to sociocultural beliefs in the general population. Psychopathology 44, 106–115. 10.1159/000319788 21196811

[B31] Pechey R. Halligan P. (2012a). Using co-occurrence to evaluate belief coherence in a large non clinical sample. PLoS ONE 7, e48446. 10.1371/journal.pone.0048446 23155383 PMC3498289

[B32] Pechey R. Halligan P. W. (2012b). Exploring the folk understanding of belief: identifying key dimensions endorsed in the general population. J. Cogn. Cult. 12, 81–99. 10.1163/156853712X633947

[B33] Poldrack R. A. Farah M. J. (2015). Progress and challenges in probing the human brain. Nature 526, 371–379. 10.1038/nature15692 26469048

[B34] Poldrack R. A. Yarkoni T. (2016). From brain maps to cognitive ontologies: informatics and the search for mental structure. Annu. Rev. Psychol. 67, 587–612. 10.1146/annurev-psych-122414-033729 26393866 PMC4701616

[B35] Quine W. V. Ullian J. S. (1970). The Web of Belief. New York, NY: Random House.

[B36] Schwitzgebel E. (2010). “Belief,” in The Stanford Encyclopedia of Philosophy, ed E.N. Zalta (Stanford, CA: Stanford University).

[B37] Seitz R.J. Paloutzian R.F. Angel H.-F. (2018). From believing to belief: A general theoretical model. J. Cogn. Neurosci. 30, 1254–1264. 10.1162/jocn_a_01292 29877765

[B38] Sperber D. (2009). Culturally transmitted misbeliefs. Behav. Brain Sci. 32, 534–535. 10.1017/S0140525X09991348

[B39] Stone T. Young A. W. (1997). Delusions and brain injury: the philosophy and psychology of belief. Mind Lang. 12, 327–364. 10.1111/1468-0017.00051

[B40] Vetter P. Newen A. (2014). Varieties of cognitive penetration in visual perception. Conscious. Cogn. 27, 62–75. 10.1016/j.concog.2014.04.007 24836978

